# Reliability of surgeon-reported MRI findings to a national spine register

**DOI:** 10.1007/s00701-025-06524-5

**Published:** 2025-04-14

**Authors:** Ole Kristian Alhaug, Håvard Furunes, Simran Kaur, Nynne Blomfeldt, Filip C. Dolatowski

**Affiliations:** 1 Orthopaedic Department, Akershus University Hospital, PO Box 1000, N- 1478 Loerenskog, Norway; 2 The Research Center for Age-Related Functional Decline and Disease, Innlandet Hospital Trust, PO Box 68, N- 2313 Ottestad, Norway; 3 Department of Surgery, Innlandet Hospital Trust, Innlandet Hospital Gjøvik, Kyrre Grepps Gate 11, 2819 Gjøvik, Norway; 4 Faculty of Medicine, University of Oslo, Oslo, Norway; 5 Vestfold Hospital Trust, Tønsberg Sykehus, Halfdan Wilhelmsens Allé 22, 3112 Tønsberg, Norway; 6 Orthopaedic Department, Martina Hansens Hospital, Dønskiveien 8, 1346 Gjettum, Norway; 7 Orthopaedic Department, Oslo University Hospital, PO Box 4956, N- 0424 Oslo, Norway

**Keywords:** Spine registry, MRI reliability, Data accuracy, Lumbar spinal stenosis, Lumbar disc herniation, Modic changes

## Abstract

**Purpose:**

Spine registries contribute to valuable knowledge and research; however, the data quality has been questioned. MRI findings are crucial for diagnostics and grading of degenerative spinal disorders. We aimed to explore the reliability of surgeon-reported MRI findings in a national spine registry (NORspine).

**Methods:**

We assessed the reliability of MRI findings from three spine centres. Two spine surgeons re-examined previously surgeon-reported MRI findings for a sample of NORspine patients. We assessed the inter-rater reliability and the reliability between the NORspine registry and each study rater by Cohen's Kappa (ƙ).

**Results:**

Two spine surgeons reassessed preoperative MRI of the lumbar spine for 276 previously enrolled NORspine patients; 92 at each treating centre equally distributed by three categories of spinal procedures: removal of disc herniation (LDH), decompression of lumbar spinal stenosis (LSS), and lumbar fusion. The inter-rater reliability varied from fair (0.21) to substantial (0.75) (most reliable for detecting LDH and LSS). The reliability between the NORspine registry and each rater varied from slight (0.13) to substantial (0.75). The highest reliability was found for LDH and LSS (ƙ = 0.72–0.75), while degenerative disc (DDD), foraminal stenosis (FS) and Modic changes had lower reliability (ƙ = 0.27–0.49).

**Conclusion:**

The reliability for surgeon-reported MRI diagnostics to the NORspine registry varied and was substantial for LDH and LSS, moderate for DDD and FS, and slight or fair for Modic changes.

## Introduction

Medical registries have gained popularity in research, and they monitor the quality and effect of treatment. However, the data quality of spine registries has been questioned, and previous audits report varying data accuracy [1, 2, 10]. Mayer et al. found high levels of inaccuracy in a German spine register in 2020 and advocated against using these data [10]. In two validation studies of the Norwegian Registry of Spine Surgery (NORspine), we have previously demonstrated the underreporting of comorbidities and complications. We found patient-recorded data more accurate than the surgeon-recorded equivalents [1, 2]. Surgeon-reported MRI diagnostics were not evaluated in the audits above.

With clinical history and examination, a lumbar spine MRI makes the foundation for diagnosing and grading spinal disorders. MRI is the gold standard in spine diagnostics, affecting clinical treatment decisions [8]. Research of spinal degenerative disorders also heavily relies on MRI findings. The reliability of MRI assessment may vary with the specific spinal condition [6, 7].

In NORspine, surgeons report the perioperative details, including MRI findings; we aimed to assess the data quality of surgeon-reported MRI assessment for a sample of previously recorded NORspine patients [11].

## Methods

This study was a cross-sectional reassessment of previously surgeon-reported NORspine MRI diagnostics. Although patient participation is voluntary, NORspine is a national compulsory register, and 39 Norwegian spine centres (must) recruit patients. The completeness of NORspine data is 80%, and 75% of the patients respond at 12 months follow-up [11]. NORspine includes patient-reported baseline data, such as patient characteristics and symptoms described by standard PROMs. Surgeons report diagnostics, including MRI findings, perioperative surgical details, and any perioperative complications. MRI reports are available to the surgeons when they report to NORspine. The following MRI variables are recorded: Modic changes (type 1 or 2), disc herniations (extraforaminal, intraforaminal, or central), degenerative disc disease (without other findings); spinal stenosis (central, lateral, or foraminal), and synovial cysts. Furthermore, patients report clinical outcomes after three and 12 months by standard PROMs (Oswestry Disability Index (ODI), Numeric Rating Scale (NRS) for back and leg pain, Global Perceived Effect (GPE) transitional scale, quality of life by EuroQol- 5 Dimension (EQ- 5D).

We had legal permission to access MRI images and electronic patient records (EPR) at three author-affiliated hospitals. At each hospital, we reassessed lumbosacral MRIs of 90–96 consecutive NORspine patients who received spinal surgery during 2021 and 2022: 30–32 disc herniation removals, 30–32 decompressions for LSS, and 30–32 spinal fusions.

The MRIs were done during regular clinical practice, and different protocols, slice-thickness, and magnets may have been used. MRI examinations consisted of sagittal T2-weighted images, sagittal T1-weighted images, sagittal fat-suppression images, and axial T2-weighted images.

Study raters are consultant orthopaedic spine surgeons. For each patient, raters reviewed the surgical record (Electronic Patient Record (EPR)) before they assessed the latest preoperative MRI examination of the lumbosacral spine; the MRI reports were available. Study raters were blinded to the original surgeon-reported findings, and their MRI diagnostics were recorded using a blank version of the standardized NORspine surgeon questionnaire.

### Statistics

We reported mean values with 95% CIs for continuous variables and numbers and proportions for dichotomous variables. Two surgeons reviewed the patient EPR and MRI examination. We report the inter-rater reliability by Cohen's Kappa (ƙ). Reliability was graded by Cohen's Kappa and categorized as follows: None (0.00–0.20), minimal (0.21–0.39), weak (0.40–0.59), moderate (0.60–0.79), strong (0.80–0.90), and almost perfect (0.91–1.00) [9]. McHugh also suggested that a kappa under 0.60 was inadequate [9]. The MRI assessment in this study differed somewhat between the raters, and we could not establish a gold standard. Hence, we could not determine the accuracy of NORspine MRI variables, only the reliability between NORspine and each rater. The calculations were done using SPSS version 26 (IBM Corp. released in 2017. IBM SPSS Statistics for Windows, version 26. Armonk, NY, USA).

We used the sample sizes reported by Bujang et al. and presumed true marginal frequencies to be the same, and aimed at detecting a difference in Kappa of 0.2 as the thresholds for interpreting Kappa have steps of 0.2 points [5, 9]. We expected a kappa of 0.5 and chose an alpha of 0.05 and a beta of 0.80; hence, the minimum sample size is 133 patients. To account for the uncertainty in the sample size calculation, we doubled this number and aimed to include 280 patients.

### Ethics

The Regional Committees for Medical and Health Research Ethics (2017/2157) and The NORspine board approved this study. The study was conducted following the Helsinki Declaration, and we report the results in line with STROBE guidelines [13].

## Results

We analyzed 276 patients; baseline data are displayed in Table 1. The mean (95%CI) age was 55.5(52.8–56.3) years, and 155 (56% were females. The mean preoperative ODI was 42.2 (40.3–44.1). Ninety (32.6%) patients had surgery for disc herniation, 92 (33.3%) for LSS, and 94 (34.1) had spinal fusion. Sixty-five (22.7%) had undergone previous spinal surgery.

**Table 1:** Baseline data and surgical details for 276 surgical spine patients

	Mean/number	95%CI/percentage
Age	54.55	52.78–56.33 (SD 14.95)
Gender female	155	56.2%
Smoking	45	16.4%
Body Mass Index (BMI)	28.2	27.5–28.8 (SD 5.15)
Preoperative ODI*	42.2	40.3–44.1 (SD 16.1)
Preoperative NRS** Back pain	6.9	6.6–7.1 (SD 2.05)
Preoperative NRS** Leg pain	6.7	6.5–7.0) (SD 2.37)
Previous spine surgery		
Yes, same level	35	12.7%
Yes, other level	19	6.9%
Yes, same and other	9	3.3%
No	213	77.3%
Type of surgery		
Disc herniation removal	90	32.6%
Decompression	92	33.3%
Fusion	94	34.1%
Surgical access		
Midline	236	85.5%
Anterior	3	1.1%
Wiltse	37	13.4%
Level		
L1 - 2	1	0.4%
L2 - 3	13	4.7%
L3 - 4	50	18.1%
L4 - 5	166	60.1%
L5-S	95	34.4%

*Oswestry Disability Index (0–100 (0 = no disability, 100 = bed bound))

**Numeric Rating Scale (0.10 (= no pain, 10 = maximal pain))

Inter-rater reliability varied for the different MRI variables (Table 2). The raters agreed minimally in recording extraforaminal disc herniation, degenerative disc disease, and lateral spinal stenosis (ƙ = 0.21–0.39). Raters recorded Modic-related variables with weak reliability (ƙ 0.42—0.56) except Modic 1 (ƙ = 0.606). Raters recorded disc herniation, central spinal stenosis, foraminal stenosis, and synovial cysts with moderate reliability (ƙ = 0.69—0.75).

**Table 2:** Interrater reliability for different MRI findings – 276 patients

MRI variable	Cohens kappa *	SE
MODIC	0.511	0.052
MODIC 1	0.606	0.060
MODIC 2	0.422	0.057
MODIC 1 in operated level	0.539	0.067
MODIC 1 in other level	0.539	0.123
MODIC 2 in operated level	0.481	0.064
MODIC 2 in other level	0.562	0.063
Extraforaminal disc herniation (DH)	0.212	0.139
Intraforaminal disc herniation (DH)	0.477	0.129
Degenerative disc only (DD)	0.390	0.130
Lumbar disc herniation (central (LDH))	0.739	0.042
Lumbar spinal stenosis (central (LSS))	0.751	0.043
Lumbar spinal stenosis (lateral (LSS))	0.361	0.056
Foraminal stenosis (FS)	0.699	0.046
Cyst	0.689	0.120

*Cohens kappa interpretation: 0.00–0.20 = “None”, 0.21–0.39 = “Minimal”, 0.40–0.59 = “Weak”, 0.60–0.79 = Moderate, 0.80–0.90 = “Strong”, and = 0.91–1.00 “Almost perfect”

The reliability between previously recorded NORspine MRI diagnostics and the present reexamination is displayed in Table 3. The reliability was minimal for Modic variables (ƙ = 0.271–0.369) except for Modic 1 at another level, which was none (ƙ = 0.129). The reliabilities for synovial cyst and lateral spinal stenosis were also minimal (ƙ = 0.299–0.365). The reliabilities for intraforaminal disc herniation, disc degeneration, and foraminal stenosis were weak (ƙ = 0.455–0.475). The reliabilities for disc herniation and central spinal stenosis were moderate (ƙ = 0.718–0.751).

**Table 3:** Reliability for NORspine vs rater 1 and 2 for different MRI findings – 276 patients

	Rater 1		Rater 2	
MRI variable	Cohens kappa *	SE	Cohens kappa *	SE
MODIC	0.369	0.054	0.404	0.052
MODIC 1	0.271	0.074	0.303	0.068
MODIC 2	0.317	0.060	0.344	0.058
MODIC 1 in operated level	0298	0.079	0.292	0.073
MODIC 1 in other level	0.129	0.106	0.182	0.110
MODIC 2 in operated level	0.283	0.070	0.324	0.068
MODIC 2 in other level	0.341	0.072	0.324	0.068
Extraforaminal disc herniation (DH)	0.325	0.174	0.438	0.206
Intraforaminal disc herniation (DH)	0.457	0.137	0.510	0.142
Degenerative disc only (DD)	0.455	0.118	0.455	0.188
Lumbar disc herniation (central (LDH))	0.718	0.043	0.764	0.040
Lumbar spinal stenosis (central (LSS))	0.751	0.043	0.717	0.046
Lumbar spinal stenosis (lateral (LSS))	0.365	0.054	0.398	0.054
Foraminal stenosis (FS)	0.475	0.053	0.494	0.057
Cyst	0.299	0.164	0.356	0.184

*Cohens kappa interpretation: 0.00–0.20 = “None”, 0.21–0.39 = “Minimal”, 0.40–0.59 = “Weak”, 0.60–0.79 = Moderate, 0.80–0.90 = “Strong”, and = 0.91–1.00 “Almost perfect”

## Discussion

We found minimal and weak reliability of MRI findings of Modic, synovial cyst, lateral spinal stenosis, intraforaminal disc herniation, disc degeneration, and foraminal stenosis in the NORspine registry except for central disc herniation and central spinal stenosis, which had moderate reliability. Furthermore, the reliability between NORspine and each rater was inferior to the inter-rater reliability.

Two previous studies reported similar variable inter-rater reliability for spine MRI findings, depending on the MRI variable examined [6, 7]. These studies reported good inter-rater reliability for certain MRI findings, including LSS, root compression, spondylolisthesis, and inferior reliability for other findings. The results from the two studies above align with our findings.

A recent study of LSS patients reported high intra- and inter-observer agreement for LSS-related MRI findings [4]. High inter-rater reliability for LSS on MRI aligns with our study; LSS-related MRI variables showed good reliability between raters, the NORspine registry, and each rater.

Inferior reliability between NORspine and each rater versus between each rater (inter-rater) could be explained by different settings for registering those variables. In clinical practice, the surgeon records MRI variables as soon as possible after completing the surgery, along with other tasks such as writing the surgeon's note, prescribing postoperative medication, and confirming sick leave. Additionally, the surgeon has to prepare the next patient. The above task adds to the registration of NORspine data and the total workload. On the contrary, in a scientific study setting, surgeons only focus on the MRI examination. We assume that a study setting enables a more thorough MRI evaluation and increases the reliability compared to the reliability of an MRI assessment performed during a busy day at the theatre.

NORspine MRI data are reported by surgeons who treated the patients. The operating surgeon may consider Some MRI findings clinically irrelevant and hence not reported to NORspine. A reassessment of MRI by study raters that did not treat the patients may, therefore, result in recording all MRI findings, irrespective of their clinical relevance.

Our findings are important when interpreting register-based MRI diagnostics. NORspine MRI findings of central spinal stenosis and disc herniation seem to be reliable. In contrast, MRI diagnostics of Modic changes, degenerative disc disease, and foraminal nerve root compression should be used cautiously and preferably crosschecked with other data sources.

Our results also underline the importance of periodically reevaluating register data's reliability, validity, and accuracy. Reducing the number of register variables could aid in increasing data reliability.

### Limitations

The inter-rater reliability was insufficient to establish a"gold standard."Hence, we did not calculate the accuracy of the MRI recordings.

The reliability of MRI findings may be affected by the quality of the MRI examination. NORspine did not register the type of magnets or MRI protocols used. A better agreement may have been achieved with standard MRI protocols [12], but on the other hand, our study represents daily practice at three Norwegian spine centres.

The authors had access to digital MRI images performed at one university hospital and two non-teaching hospitals. However, the patient baseline characteristics of our sample did not differ from the total NORspine population or a standard spine population [11]. The distribution of the main surgical procedures, one-third of disc herniation removal, one-third of spinal stenosis decompression, and one-third of spinal fusion, were constructed for this study and were not representative of a mean NORspine population. The proportion of fusion procedures is somewhat higher in our study population, and it was selected to ensure enough patients in each treatment category. Because disc herniations and central stenosis showed the best reliability in our reexamination of lumbar MRIs, and because our population had more fusion patients than the mean NORspine population, we might report a "worst case scenario" regarding the reliability of surgeon-reported MRI diagnostics.

Furthermore, the study population was not randomly selected, but we included consecutive patients. This method of selection may limit the generalizability of this study. Additionally, each registry may include different populations, record different variables, and have different strategies for collecting data, so our findings may not be generalizable to other spine registries. However, our findings point to a typical challenge with data collection that is rarely reported in register studies. Since many publications are based on registry data, we think the data quality challenge is general, and we encourage other spine registries to assess the data quality periodically.

Sample size calculations for reliability studies are challenging. The prevalence of the variable of interest affects the sample size. NORspine MRI data are reported as dichotomous variables. We used two raters and compared NORspine data to each rater; hence, the reliability calculations are based on two-by-two tables. To account for uncertainty in the power calculations, we doubled the size of the study.

The interpretation of Kappa values can be discussed. The true distribution of the MRI findings is valuable in interpreting the Kappa value, but the true distribution is left unknown in our study [3]. Furthermore, several different classification systems interpret Kappa values.

## Conclusions

Interpreting lumbar MRI findings describing degenerative spinal disease in spine registries should be done cautiously because the reliability is mostly minimal or weak. Only disc herniation and central spinal stenosis have moderate reliability, as assessed by MRI. One should consider using additional data sources when register-based MRI diagnoses are used to study degenerative spinal disease.

## Data Availability

No datasets were generated or analysed during the current study.
